# Face–Name Associative Memory Performance Among Cognitively Healthy Individuals, Individuals With Subjective Memory Complaints, and Patients With a Diagnosis of aMCI

**DOI:** 10.3389/fpsyg.2020.02173

**Published:** 2020-09-09

**Authors:** Constantinos Kormas, Ioannis Zalonis, Ioannis Evdokimidis, Elisabeth Kapaki, Constantin Potagas

**Affiliations:** First Department of Neurology, Faculty of Medicine, Aeginition Hospital, National and Kapodistrian University of Athens, Athens, Greece

**Keywords:** associative memory, face–name associations, subjective memory complaints, amnestic mild cognitive impairment, pre-clinical Alzheimer's disease, Greek version of the Face–Name Associative Memory Examination (GR-FNAME12)

## Introduction

Alzheimer's disease (AD) is a neurodegenerative disease characterized by cognitive and behavioral disturbances with insidious initiation and gradual deterioration that significantly affect patients' quality of life (Castellani et al., 2010). AD is the most frequent cause of dementia in developed countries (Kalaria et al., 2008). Because AD is a major public health problem with strong psychosocial and economic implications, early diagnosis is a main research challenge (Zhu and Sano, 2006; Maresova et al., 2015). Recent findings indicate that the neuropathophysiological processes of AD appear several years before the first clinical symptoms are observed (Price and Morris, 1999; Sperling et al., 2012). Therefore, having a reliable and validated diagnosis in the early pre-clinical phases of AD is an important clinical aim in integrated preventive disease management for asymptomatic patients.

Recent studies have proposed that biomarker tests with a higher predictive value among asymptomatic cognitively normal individuals are the measurement of amyloid-β and t-tau or p-tau in cerebrospinal fluid (CSF) and/or the amyloid-β positron emission tomography (PET) imaging (Blennow et al., 2010; Herholz and Ebmeier, 2011; Olsson et al., 2016). In more detail, clinical studies have demonstrated that the decreased CSF Aβ42 (Skoog et al., 2003; Gustafson et al., 2007; Stomrud et al., 2007), the combined high CSF t-Tau or p-Tau and low CSF Aβ42 (Soldan et al., 2016), and the higher CSF t-Tau with PiB-PET retention (*in vivo* amyloid load) (Dumurgier et al., 2017) are strongly associated with future cognitive decline among normal older adults. Despite the high clinical value of neuropathological biomarkers for the evaluation of AD, early diagnosis of the disease remains probable and is based on clinical symptoms such as memory disorders or other cognitive and behavioral changes (Biagioni and Galvin, 2011). In this context, neuropsychological evaluation plays an important role (Chapman et al., 2010). However, most traditional neuropsychological tests are designed to detect cognitive deficits at the level of clinical manifestation of AD, and they have only a limited ability to detect early and subtle cognitive disturbances in the pre-clinical period (Rentz et al., 2013). Recent work has sought in response to develop new neuropsychological measures, known as cognitive stress tests (Loewenstein et al., 2017). These tests are more demanding in that they seek to minimize the impact of cognitive reserve (CR) on individuals' performance by significantly limiting the implementation of possible compensatory strategies. The development of such neuropsychological measurements and their introduction into clinical practice would save considerable resources, reducing the need for costly and time-consuming diagnostic interventions.

Episodic memory decline is the major neurocognitive deficit in AD. Memory deficits in AD patients are characterized by significant difficulty in delayed recall and recognition of newly acquired information (Dubois and Albert, 2004; Graham et al., 2004; Rémy et al., 2005). Recent studies have established that the mechanisms of associative memory, i.e., the ability to encode and recall relationships between unrelated items, are significantly affected in the early stages of the disease (Parra et al., 2010; Della Sala et al., 2012). A typical example of associative memory in everyday life is the recall of face–name pairs. It has been empirically established in a number of studies that the specific capacity to recall names for individuals is significantly affected in AD patients (Swainson et al., 2001; Fowler et al., 2002; Kazui et al., 2005; Werheid and Clare, 2007). An experimental study investigated the ability to recall names for persons and names for objects while administering traditional neuropsychological tests to a group of high-risk individuals who did not meet the standard AD diagnostic criteria (Semenza et al., 2003). Seven months later, a subset of these patients was found to manifest clinical signs of AD. This subgroup was compared to the other participants sampled in their scores on the tests initially administered. The results showed that only scores in face–name recall were lower for the subgroup of patients who eventually presented AD.

The need for the construction of a standardized and psychometric valid neuropsychological measure for the assessing of face–name associative memory has been recognized. This gap was filled by the construction of the Face–Name Associative Memory Examination (FNAME) (Amariglio et al., 2012b) and its short form FNAME12 (Papp et al., 2014). This was followed by the creation of versions of the test for Spanish-speaking (Alegret et al., 2015), Czech-speaking (Mazancova et al., 2017), and Greek-speaking (Kormas et al., 2018) populations. An important feature of the test is that it is based entirely on the examination of the multisensory mechanisms of associative memory, which have been found to be particularly sensitive in the early stages of AD (Blackwell et al., 2004; Parra et al., 2010); this is the case with face–name memory, for example (Clare et al., 2002; Werheid and Clare, 2007). Experimental neuroimaging studies have shown that neuronal activity is reduced during the administration of FNAME to sampled participants, who, although they did not show clinical symptoms of AD disease, had shown amyloid depositions (Sperling et al., 2009; Vannini et al., 2012). Another neuroimaging study, using positron emission tomography (PET) investigated the correlation between FNAME performance and amyloid load in cognitively healthy individuals (Rentz et al., 2011). It was found that performance on the FNAME test was significantly correlated with amyloid load in the frontal cortex, posterior precuneus, posterior cingulate cortex, and lateral parietal cortex. The results also showed that this association only appeared in the FNAME test and was not found in the Selective Reminding Test which was also administered to the participants. In particular, a significant correlation in beta-amyloid load deposition was found only with the recall of face–name pairs, not with that of face–occupation pairs. In addition, a recent neuroimaging study using F-Florbetaben PET in individuals with subjective memory complaints (SMCs) showed a significant correlation between total burden of beta-amyloid deposition and the neuropsychological measurement of associative face–name memory but not of face–occupation or on the Wechsler Memory Scale-III (Sanabria et al., 2018). Finally, it has been empirically documented that the FNAME test highly sensitively differentiates patients with aMCI from cognitively healthy individuals without producing ceiling effects, as is the case of other memory measures, making it useful for the detection of the early changes that characterize the pre-clinical phases of AD.

This study compared performance among cognitively healthy individuals, individuals with SMCs, and patients with an aMCI diagnosis using the Greek version of the FNAME12 (GR-FNAME12). In addition, possible differentiation in the success rates for delayed recall performance within the SMC group and the aMCI group on two different memory modalities test (verbal and cross verbal–visual) was investigated.

## Methods

### Participants

A total of 120 participants were recruited as the research sample, which was then divided into three subgroups: (a) the control group: 70 participants (40 women) aged 70–79 years (*M* = 73.90, SD = 6.06), with 6–16 years of formal education (*M* = 11.87, SD = 3.31) and an average score on the Mini Mental State Examination (MMSE) of 29.65 (SD = 1.10); (b) individuals with SMCs: 21 participants (11 women), aged 69–80 years (*M* = 71.14, SD = 6.15), with 6–17 years of formal education (*M* = 12.42, SD = 2.96) and an average score on the MMSE of 29.14 (SD = 1.28); and (c) patients with a neurological diagnosis of aMCI: 29 participants (18 women), aged 68–81 years (*M* = 73.20, SD = 7.96), with 6–18 years of formal education (*M* = 11.03, SD = 4.80) and an average score on the MMSE of 25.38 (SD = 2.96). One-way analysis of variance (ANOVA) showed that the subgroups did not differ significantly in age [*F*_(2, 119)_ = 1.422, *p* = 0.245] or education [*F*_(2, 119)_ = 0.944, *p* = 0.392].

The control group was made up of volunteers from Athens, the capital city of Greece. The inclusion criteria were (1) absence of a history of neurological or psychiatric problems, traumatic brain damage, alcohol or drug abuse, or visual disturbance, as reported by participants; (2) MMSE score ≥27; and (3) no report of subjective cognitive complaints. The participants of the two clinical subgroups were recruited by the Memory Clinic of the National and Kapodistrian University of Athens at Aeginition Hospital. The inclusion criteria for the group of SMCs were as follows: (1) older than 50 years old, (2) at least 6 years of formal education, (3) previous visit to the Memory Clinic on the grounds of a subjective complaint of memory impairment, (4) MMSE score ≥27, (5) normal scores based on normative data in the neuropsychological memory measures of the Greek Verbal Learning Test (GVLT), and (6) the absence of neurological and/or psychiatric history. The inclusion criteria for the aMCI group of patients were as follows: (1) neurological diagnosis of aMCI based on the Petersen's criteria (Petersen et al., 2014), (2) aged over 50 years, (3) at least 6 years of official education, (4) MMSE score of 23–26, (5) *z* score ranging between −1.00 and −2.00 in the GVLT, and (6) absence of neurological and/or psychiatric history. The ethics committee for the Aeginition Hospital approved the study protocol, which followed the principles outlined in the Declaration of Helsinki. All participants were informed of the purpose of the study. They then signed a written informed consent form before they could participate in the study.

### Procedure and Neuropsychological Measures

Each patient was individually evaluated during the morning hours in the Memory Clinic of the First Neurology Department of the National and Kapodistrian University of Athens at Aeginition Hospital. The evaluation of global cognitive status was conducted using the MMSE (Folstein et al., 1975), which was standardized in Greek (Fountoulakis et al., 2000); delayed free recall was measured using the GVLT (Vlahou et al., 2013), which is based on the California Verbal Learning Test (Delis et al., 1987), for testing the verbal memory and the GR-FNAME12 for testing the cross visual–verbal associative memory domain (Kormas et al., 2018) (Table 1).

**Table 1:** Demographic characteristics and performances on the neuropsychological measures of the three subgroups as mean (standard deviation).

	**Controls** **(*N* = 70)**	**SMC** **(*N* = 21)**	**aMCI** **(*N* = 29)**
Age, years	73.90 (6.06)	71.14 (6.15)	73.20 (7.96)
Formal education	11.87 (3.31)	12.42 (2.96)	11.03 (4.80)
MMSE, range score 0–30	29.65 (1.10)	29.14 (1.28)	25.38 (2.96)
GVLT-Delayed recall, range score 0–16	12.21 (3.15)	11.33 (2.83)	7.87 (4.20)
GR-FNAME12-Delayed recall, range score 0–12	4.83 (2.32)	3.33 (2.08)	1.93 (0.75)

*aMCI, patients with amnestic mild cognitive impairment diagnosis; GR-FNAME12, Greek version of the Face–Name Associative Memory Examination; GVLT, Greek Verbal Learning Test; MMSE, Mini Mental State Examination; SMC, cognitively healthy individuals with subjective memory complaints*.

### Statistical Analysis

The statistical analyses of data were performed using SPSS23 (IBM Corp., released 2015, IBM SPSS Statistics for Windows, Version 23.0, IBM Corp., Armonk, NY). Initially, the comparison of the performance between the three subgroups in the GR-FNAME12 test was examined with one-way ANOVA. Finally, it was examined whether the two clinical subgroups of the sample exhibited different mnemonic capabilities in tests of different modalities by comparing the success rates for each memory test. The raw scores for GVLT and GR-FNAME12 were converted to rates of success with a common percentile system, according to the following procedure: (1) for GVLT (raw score/16) ^*^ 100 and (2) for GR-FNAME12 (raw score/12) ^*^ 100. Subsequently, paired analyses with the *t*-test were performed to compare the success rates of free delay performance between the two memory tests for the subjective complaints group and the aMCI group.

### Data Analysis

#### Delayed Recall Performance Among the Three Groups in the Face–Name Associations [Greek Version of the Face–Name Associative Memory Examination (GR-FNAME12)]

The results of the one-way ANOVA showed significant differences in delayed recall performance among the three subgroups in the GR-FNAME12 [*F*_(2, 119)_ = 22.204, *p* < 0.001]. Bonferroni *post hoc* analyses revealed the following relationships among scores (a) control group (*M* = 4.83, SD = 2.32) > SMC group (*M* = 3.33, SD = 2.08) [95% CI = −2.6884 to −0.3116, *p* = 0.009], (b) control group > aMCI group (*M* = 1.93, SD = 0.75) [95%CI = −3.9548 to −1.8452, *p* = 0.001], and (c) SMC > aMCI patients group [95%CI = −2.7686 to −0.0314, *p* = 0.043].

#### Delayed Recall Performance Among the Three Groups in the Word List [Greek Verbal Learning Test (GVLT)]

The results of the one-way ANOVA indicated significant differences in delayed recall performance among the three groups for the GVLT [*F*_(2, 119)_ = 16.993, *p* < 0.001]. The Bonferroni *post hoc* test demonstrated a significantly lower performance for the aMCI group (*M* = 7.87, SD = 4.20) than in the control group (*M* = 12.21, SD = 3.15) (95% CI = −6.1133 to −2.5667, *p* = 0.001) and the SMC group (*M* = 11.33, SD = 2.83) (95% CI = −5.7608 to −1.1592, *p* = 0.001). By contrast, no significant difference was observed between the control group and the SMC group (95% CI = −2.8779 to 1.1179, *p* = 0.549).

#### Comparison of Success Percentage Rates of Delayed Recall Performance in the Two Memory Tests

##### Cognitively healthy individuals with subjective memory complaints (SMC group)

The results of a paired *t*-test [*t*_(20)_ = 7.957, *p* < 0.05] showed significant differences in success rates across different modalities of delayed recall measures: GVLT (*M* = 70.83, SD = 17.71) > GR-FNAME12 (*M* = 27.78, SD = 17.35).

##### Patients with amnestic mild cognitive impairment diagnosis (aMCI group)

Similarly, in the aMCI group, a significant difference was observed between success rates for the different measurements of delayed recall, as determined by a paired *t*-test [*t*_(28)_ = 5.743, *p* < 0.05]; GVLT (*M* = 46.34, SD = 25.55) > GR-FNAME12 (*M* = 17.53, SD = 8.77).

## Discussion

In this study, we investigated performance differences among cognitively healthy individuals, individuals with SMCs, and patients with aMCI diagnoses in the GR-FNAME12. It was found that the three groups differed significantly in their delayed performance recall of face–name associations. The group of cognitively healthy individuals had the highest performance, and the patients with aMCI had the lowest, and the scores of individuals with SMCs were intermediate between the other two. These results demonstrate that delayed recall of face–name pairs sufficiently differentiates the performance of cognitively normal individuals from pre-clinical subgroups, which is similar to the results of previous studies (Rentz et al., 2011; Papp et al., 2014). A significant finding of particular clinical value was that GR-FNAME12 sufficiently separates the performance of the normal group from that of cognitively healthy individuals with SMCs, confirming the significant correlation that has previously been found between performance in face–name associations and neuropathological changes during the pre-clinical stages of AD (Sanabria et al., 2018). However, the GVLT memory test failed to distinguish the performance between the normal group and the SMC group. On this evidence, it seems that the delayed recall of word lists is not a sufficiently sensitive neuropsychological measure for detecting subtle memory changes in the pre-clinical stages of AD, as has been reported from previous studies (Rentz et al., 2011).

It was also found that individuals with SMCs and patients with aMCI exhibit significantly higher success rates for the delayed recall of a word list (GVLT) than for the recall of face–name pairs (GR-FNAME12). It appears that the retrieval of a single verbal stimulus is accomplished through semantic networks and conceptual categorization strategies that also contribute to the recall of newly acquired material with higher success rates (Cherry et al., 2012). However, the recall of face–name associations appears to be a particularly demanding cognitive process, requiring the binding of two distinct aspects of information into a coherent unit that is not supported by previous conceptual links (Craigie and Hanley, 1997), and thus the specific modular memory function is particularly vulnerable to clinical disturbances at the early stages of AD progression (Werheid and Clare, 2007).

If aging is to be approached as a continuum between normal and pathological decline, clinicians need reliable and sensitive assessment measures that can detect the cognitive deficits indicative of the initial stages of AD in a timely manner. It must be a clinical and research priority to design neuropsychological measurements that have sufficient sensitivity and specificity (Weissberger et al., 2017) and are associated with typical AD symptoms to improve the chances of early detection and diagnosis. If this is achieved, a significant increase can be expected in the likelihood of early interventions intended to maintain the quality of life among patients. Early preventive treatment of asymptomatic patients could prolong their autonomy and independence in their social, family, and working environments.

Difficulties in face–name associative memory are among the most frequent memory complaints reported by the elderly. The FNAME associative memory examination offers higher ecological validity in this context (Zelinski and Gilewski, 1988; Leirer et al., 1990) than many other neuropsychological measurements and can support adequate response to the requirements of high-level analytical neuropsychological examination. The administration of this test can lead to significant clinical benefits, as it is considered particularly sensitive for the detection of early AD deficits, an observation supported by the findings of recent studies (Rentz et al., 2011; Sanabria et al., 2018), which show significant cortical correlations between the recall performance for face–name pairs and amyloid deposition in the frontal and posterior cortical areas in both cognitively healthy individuals and asymptomatic individuals with SMCs. However, the results of neither the face–occupation subscale of FNAME, the Selective Reminding Test, nor the Wechsler Memory Scale-III were found to be significantly correlated with amyloid deposition in the above investigations.

Rentz et al. (2017) argued that the delayed recall of face–name associations is particularly demanding, as it includes the binding of two types of information in an arbitrary manner and in the absence of conceptual support (Werheid and Clare, 2007). Therefore, the retrieval of appropriate names in relation to faces is more difficult than that of occupations or common nouns, as names do not provide semantic information, while other types of words and biographical information can be classified in relation to a semantic classification and are easily associated with conceptual characteristics (Fogler and James, 2007; Amariglio et al., 2012a). The retrieval of face–name pairs has greater requirements than that of other memory domains (McWeeny et al., 1987; Cohen, 1990). The inability to recall face–name pairs has attracted interpretation in the context of a broader weakness in the associative memory mechanisms. The associative-deficit hypothesis (Naveh-Benjamin, 2000, 2015) holds that both elderly and patients in the early stages of AD have considerable difficulty in merging different aspects of an episode into a coherent unit. In this sense, the glue that links the various aspects (characteristics) of an episode is simply not sufficiently effective. Therefore, although each of the ingredients can be satisfactorily maintained, the associations that link the properties of the single item of information are weakened, and the difficulty of retrieving the combined information is thus reduced. The synergistic deficit appears to have been triggered by the weakening of automatic binding processes and the inability to implement complex encoding and recall strategies due to pathological changes in the prefrontal and temporal areas of the brain.

Finally, the finding that both individuals with SMCs and patients with aMCI have significantly higher success rates in retrieving a word list than in delayed recall of face–name pairs could be interpreted in terms of neuropathoanatomical correlations. More specifically, the activation location of neural structures into the temporal lobe depends on the specific requirements of the given memory task (Mitchell et al., 2000; Sperling et al., 2001). Associative memory tasks (including face–name pairing) require the encoding of new associations between irrelevant stimuli and activate the anterior areas of the hippocampus formations CA 2 and CA 3 and the dentate gyrus (Zeineh et al., 2003). By contrast, the activation of the posterior areas of the hippocampus is related to the memorization of single information units, such as words (Brewer et al., 1998; Wagner et al., 1998; Kirchhoff et al., 2000). It has been established that anterior areas in the hippocampus share a large number of connections with cortical areas (Moser and Moser, 1998). The main entrance from the multisensory cortical areas to the hippocampus takes place in the anterior areas by the entorhinal cortex, known as the perforant pathway (Van Hoesen, 1982). Pathological changes in the perforant pathway are already evident at the early stages of AD (Hyman et al., 1984) and can result in a significant impairment of the associative memory processes as early as the pre-clinical stages of AD.

Previous research have found that the face–name associative memory performance was not affected by education level in contrast to traditional memory measures (Werheid and Clare, 2007; Amariglio et al., 2012a; Alegret et al., 2015; Kormas et al., 2018). Highly educated individuals are characterized by rich semantic networks, and they have the ability to develop advanced and sophisticated conceptual strategies that positively contribute to the recall of word lists and/or stories (O'Shea et al., 2018). In this context, clinical studies have found that higher education improves CRs (Thow et al., 2018), and individuals with higher levels of CR are able to compensate a higher degree of brain dysfunction (Corral et al., 2006). Consequently, greater CR strongly influences the performance on neuropsychological measures at the stage in prodromic cognitive dysfunction (Weintraub et al., 2018). However, it has been proposed that face–name memory has specific distinguishing features from other mnemonic phenomena (Werheid and Clare, 2007). People names are characterized by low-frequency production, unlimited phonological combinations, meaninglessness, arbitrary connection to the faces, inability of visualization, and lack of multiple semantic associations with the faces (Cohen, 1990; Burton and Bruce, 1992; Cohen and Burke, 1993; Craigie and Hanley, 1997; Carson et al., 2000). Therefore, the recall of face–name associations not being affected by semantic networks or by the development of conceptual strategies, as a consequence, higher level of education and greater CR do not influence the face–name memory performance (Rentz et al., 2013; Loewenstein et al., 2017). These features may make the face–name associative memory measure particularly sensitive to early and subtle memory dysfunction at pre-clinical stages. The present study has some limitations. First, this was a cross-sectional study, and its data reflect task performance at a certain point in time. In addition, no standardized psychometric scale was used to determine the features of the group with SMCs. Finally, neuroimaging data and biomarkers for the sampled participants were not assessed, and as a result, the participants could not be investigated in terms of correlation with performance on the GR-FNAME12.

In conclusion, the results show that GR-FNAME12 sufficiently distinguishes performance among cognitively health individuals, individuals with subjective complaints of memory disturbances, and patients with an aMCI diagnosis. Bearing in mind that the GR-FNAME12 is an innovative neuropsychological measure of high ecological validity, designed to detect subtle memory declines in the pre-clinical period of AD, its construction is needed to fill a significant gap in Greek medical assessment of cognitive decline. This study supports the clinical validity of GR-FNAME12, and its administration should be encouraged as part of neuropsychological protocols to meet clinical and research needs.

## Data Availability Statement

The raw data supporting the conclusions of this article will be made available by the authors, without undue reservation.

## Ethics Statement

The ethics committee of the Aeginition Hospital reviewed and approved the study protocol, which followed the principles outlined in the Declaration of Helsinki. The patients/participants provided their written informed consent to participate in this study.

## Author Contributions

CK contributed to study concept and design, acquisition of data, analysis and interpretation of data, and drafting of the manuscript. IZ, IE, EK, and CP contributed to study concept and design and critical revision of the manuscript for important intellectual content. All authors contributed to the article and approved the submitted version.

## Conflict of Interest

The authors declare that the research was conducted in the absence of any commercial or financial relationships that could be construed as a potential conflict of interest.
